# Marine-derived polysaccharides: the potential agents against neurodegenerative diseases

**DOI:** 10.3389/fphar.2024.1506789

**Published:** 2024-12-18

**Authors:** Lin Zhu, Yuqian Ren, Shenghan Zhang, Yunliang Guo, Jinbao Zong, Yingjuan Liu

**Affiliations:** ^1^ Clinical Laboratory, Qingdao Traditional Chinese Medicine Hospital, Qingdao Hiser Hospital Affiliated of Qingdao University, Qingdao Key Laboratory of Immunodiagnosis, Qingdao, China; ^2^ Shandong Provincial Key Laboratory of Pathogenesis and Prevention of Neurological Disorders, Shandong Provincial Collaborative Innovation Center for Neurodegenerative Disorders, Institute of Brain Science and Disease, Qingdao University, Qingdao, China

**Keywords:** neurodegenerative diseases, polysaccharides, marine, fucoidan, chitosan

## Abstract

Neurodegenerative diseases are primarily characterized by the selective loss of neurons in the brain, leading to a significant and widespread global public health burden. Although numerous mechanisms underlying neurodegenerative diseases have been elucidated, effective therapeutic strategies are still being explored. Several drugs have been proposed to halt disease progression; however, they often come with severe side effects. Recently, polysaccharides have garnered considerable attention due to their antioxidant, anti-neuroinflammatory, anticholinesterase, and anti-amyloidogenic properties. The ocean contains a large number of animals, plants, algae and fungal species. Its rich sources and wide availability make the research on marine drugs become a hot topic. Recently, polysaccharides dominated by fucoidan and chitosan have been reported to inhibit the progression of neurodegenerative diseases in a variety of ways. In this review article, we provide a comprehensive summary of reported polysaccharides that intervene in neurodegenerative diseases with the aim of exploring their potential as therapeutic agents.

## 1 Introduction

Neurodegenerative diseases (NDDs) arise from the progressive loss of neurons or their myelin sheathing and involve a fundamental role of neuroinflammation in the pathophysiology, resulting in functional impairment over time (Heemels, 2016; Agnello and Ciaccio, 2022). The prevalence of NDDs is extensive and encompasses Alzheimer’s disease (AD), Parkinson’s disease (PD), Huntington’s disease (HD), multiple sclerosis (MS), amyotrophic lateral sclerosis (ALS), traumatic brain injury (TBI)and age-related macular degeneration (AMD). These disorders may have genetic, environmental, or multifactorial etiologies (Höglund and Salter, 2013; Temple, 2023). The alterations in markers of inflammation could initiate or exacerbate neuroinflammation and perpetuate the neurodegenerative process (Furman et al., 2019; Tansey et al., 2022). ND affects individuals across all age groups and imposes substantial economic burdens on patients and their families (Nichols et al., 2022; Nandi et al., 2022). To date, no effective cure has been identified for any neurodegenerative disease (Heemels, 2016; Agnello and Ciaccio, 2022). Currently, drug therapy dominates the treatment landscape for neurodegenerative diseases. However, challenges such as drug toxicity, high costs, limited availability of drugs, and the emergence of drug resistance persist. Therefore, there is a pressing need to develop novel therapeutic agents.

**FIGURE 1:**
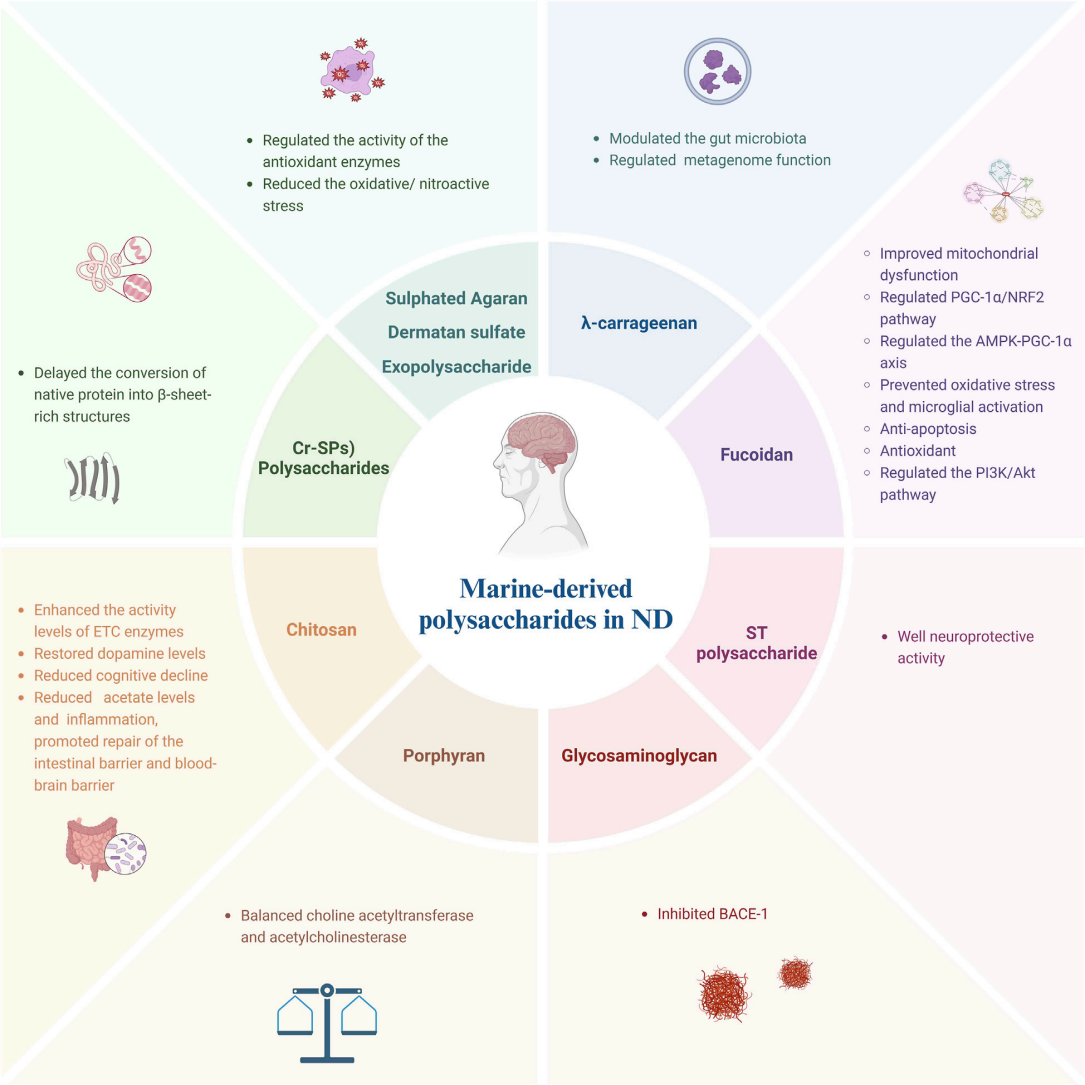
Polysaccharides derived from marine against neurodegenerative disease.

Marine species exhibit remarkable diversity. China has been at the forefront of utilizing marine resources for medicinal purposes (Fu et al., 2016). In recent years, significant emphasis has been placed on developing new drugs and products derived from marine sources within pharmaceutical research (Hu et al., 2011). Marine drugs encompass various categories including polysaccharides, peptides, terpenoids, steroids, tannins, flavonoids and other t compounds (Hu et al., 2011). Polysaccharides are widely distributed in nature. Marine polysaccharides are a kind of polysaccharides isolated and purified from Marine and lake organisms (Wang et al., 2012). Based on their sources, marine polysaccharides can be categorized into three types: animal polysaccharides, plant polysaccharides, and microbial polysaccharides (Wang et al., 2012). Polysaccharides exhibit various effects such as anti-aging properties, antioxidant activity, ulcer prevention abilities, virus inhibition capabilities,cancer-fighting potential, hypoglycemic effects,and regulation of body immunity (Wang et al., 2012; Yu et al., 2018; Zeng et al., 2019; Jiang et al., 2021; Zhou et al., 2022). This review provides a comprehensive summary of recent advancements in utilizing marine polysaccharides for the treatment of neurodegenerative diseases as shown in Figure 1.

## 2 Polysaccharides from animals

### 2.1 Chitosan

Chitosan, a biopolymer derived from chitin found in the exoskeleton of crustaceans such as shrimp and crabs (Ouyang et al., 2017; González-Chavarría et al., 2023), has gained significant attention across various fields due to its biocompatibility, biodegradability, and non-toxic nature (Ouyang et al., 2017; Thirupathi et al., 2022; Budai et al., 2023). Chitosan has been investigated for its potential role in the management and treatment of neurodegenerative diseases by scavenging free radicals and reducing oxidative stress (Ahlawat et al., 2020; Pramod Kumar and Harish Prashanth, 2020; Etxebeste-Mitxeltorena et al., 2024). Chitooligosaccharides attenuate Cu^2+^-induced cellular oxidative damage and cell apoptosis involving Nrf2 activation (Huang et al., 2015), Chitosan, contributed to the attenuation of oxidative damage and could be used as a nutritional agent for AD treatment. Additionally, both chitosan (CHT) and its quarternary derivative N-trimethyl chitosan chloride exhibited a concentration-dependent inhibiting activity on Aβ 40 fibrillogenesis mainly depending on the attractive electrostatic interactions between the positively charged moieties in chitosan and the negatively charged residues in Aβ40 (Liu et al., 2015). Several animal studies have suggested that chitosan has the potential to enhance cognitive function and memory, which could be beneficial for conditions such as AD (Amini and Abdolmaleki, 2022; Saleem et al., 2022; Yang et al., 2023b). Following a 10-day induction with rotenone (500 μM) in male adult flies, the administration of low molecular weight chitosan demonstrated improvements in *drosophila* dyskinesia, exploratory defects, and increased survival up to 16 days (Pramod Kumar and Harish Prashanth, 2020). Chronic inflammation is recognized as a contributing factor in NDDs (Yang et al., 2022). Chitosan has demonstrated anti-inflammatory effects that may help alleviate neuroinflammatory processes associated with these conditions (Yang et al., 2018; Mohebichamkhorami et al., 2023; Rajkumar et al., 2024). It could modulate inflammatory mediators in lipopolysaccharides (LPS)-stimulated BV2 microglia via the MAPK signaling pathway, which was negative to its molecular weight, with lower molecular weight showed higher activity (Pangestuti et al., 2011). Wang et al. administered chitosan to MPTP-induced Parkinson’s disease mouse models which resulted in reduced inflammation along with effective protection against dopamine neuron damage while improving motor symptoms (Wang et al., 2024).

The blood-brain barrier (BBB) is the block of the delivery of drugs. As a non-toxic, safe, and potential agent, chitosan has attracted attention for use in drug delivery systems. Efficient delivery of dopamine (DA) is crucial for Parkinson’s disease treatment but pure dopamine cannot penetrate BBB due to its hydrophilicity (De Virgilio et al., 2016). Ren et al. (2017) highlighted chitosan hydrogel as a promising carrier system for drug delivery since their synthesized injectable quaternized chitosan hydrogel exhibited no cytotoxicity while effectively enabling local release of DA along with anti-inflammatory drugs. Hassan et al. (2024) confirmed significant anti-Parkinson’s and antidepressant effects achieved through chitosan-coated-Tanshinone IIA-nanostructured lipid carriers compared to uncoated counterparts in rotenone-induced PD rat models. Yang et al. discovered that Res-loaded CS/TPP nanoparticles can effectively penetrate the blood-brain barrier and inhibit microglia activation, thereby regulating brain glucose homeostasis, oxidative stress, and neuroinflammation (Yang et al., 2023a). Insulin exhibits a potent therapeutic effect in the treatment of Alzheimer’s disease (AD), and intranasal delivery of insulin represents an effective treatment strategy. Notably, chitosan Transfersulin nanovesicles prepared through thin film hydration have been demonstrated to enhance the neuroprotective efficacy on the hippocampus of diabetic model rats by facilitating continuous intracellular drug uptake and mediating insulin transport to the brain. This study highlights their potential as a promising approach for AD therapy (Bedse et al., 2015; Nojoki et al., 2022). The neuroprotective potential of chitosan-based FTY720 nanoformulation for preventing Parkinson’s disease (PD) through *p*P2A-mediated epigenetic regulation was investigated by Sardoiwala et al. (2021). It facilitates the transport of therapeutic agents across the BBB, potentially enhancing the efficacy of drugs used in treating NDDs (Ren et al., 2017; Zhang L. et al., 2020; Omidian et al., 2024). While there is promising research on chitosan’s potential in addressing neurodegenerative diseases, much of the work is still in the preclinical or early clinical stages. More extensive human trials are required to fully understand its benefits, optimal dosages, and mechanisms of action. The application of this native polysaccharide is limited by its high molecular weight and highly viscous nature resulting in its low solubility in acid-free aqueous media.

### 2.2 Glycosaminoglycan

Glycosaminoglycans play a pivotal role in the regeneration of the mammalian CNS. Glycosaminoglycan, especially those coating the surface of endothelial cells and leukocytes, regulate some of the major events during the inflammation process by which exogenous sulfated structures downregulating inflammation processes (Morla, 2019). As the rate-limiting step in Aβ production, the β-site amyloid precursor protein cleaving enzyme 1 (BACE-1) has emerged as a key drug target of AD (Prati et al., 2018; Monteiro et al., 2023). Previous studies have reported that heparin, a glycosaminoglycan, exhibits significant inhibitory effects on BACE-1 activity. However, the high anticoagulant potential limits heparin’s suitability as a therapeutic biomolecule. Therefore, alternative sources with reduced anticoagulant properties have garnered more attention (Engelberg, 2004; Alavi Naini and Soussi-Yanicostas, 2018; Yang et al., 2021). Notably, chondroitin sulfate extracted from *Placopecten magellanicus* containing a high content of 6-sulphated N-acetyl glucosamine demonstrates potent inhibition against BACE-1 (IC50 = 4.8 μg/mL), suggesting a more favorable therapeutic profile (Mycroft-West et al., 2023). Additionally, a marine-derived glycosaminoglycan containing heparan sulphate from *Portunus pelagicus*, was identified to inhibit human BACE1. Importantly, the interactions between *P. pelagicus* glycosaminoglycan and BACE1 differ significantly from those observed with heparin (Mycroft-West et al., 2019). Further investigations are warranted to elucidate the underlying mechanisms. Besides, de Sousa et al., obtained a type of Glycosaminoglycans, dermatan sulfate from the *ascidian Phallusia* nigra and demonstrated its neuroprotective and antioxidant properties in rotenone induced Neuro-2A cells. The underlying mechanisms probably resulted from regulating the activity of the antioxidant enzymes superoxide dismutase and catalase, leading to the decreased levels of lipid peroxidation to protected cells from damage (de Sousa et al., 2020). Recently, *Phallusia nigra* dermatan sulfate (PnD2,6S) was also reported with neuritogenic and neuroprotective role. It had a better neuritogenic effect than chondroitin sulfate and dermatan sulfate at a lower concentration (0.05 μg/mL) in pesticide rotenone induced a neuro 2A murine neuroblastoma cell line, suggesting the sulfation pattern was important for neuritogenic activity (Medeiros et al., 2023).

## 3 Polysaccharides from algae

Generally, the chemical composition of polysaccharides from microalgae and macroalgae is very complex and in high variance depending on the algal source, harvesting time, cultivation method. Due to the huge biodiversity and wide application in foods and folk medicine, seaweeds are considered as an attractive source of bioactive compounds. Many bioactive compounds, partially purified polysaccharides have been tested for various therapeutic activities against various human diseases (Pradhan et al., 2020).

### 3.1 Fucoidan

Fucoidans, abundant in the brown seaweeds and other marine species, are a group of sulfated polysaccharides composed of fucose, other monosaccharides (mannose, galactose, glucose, xylose, etc.) and uronic acids, even acetyl groups and proteins (Luthuli et al., 2019). Despite its complex structure, repeated (1→3)-l-fucopyranose, or alternating and repeated (1→3)- and (1→4)-l-fucopyranose, are recognized as the structural backbone. C-2 and C-4 on fucosyl residues could be substituted with sulfate and/or acetate groups (Zahan et al., 2022). Fucoidans derived from different sources possess variations in structural characteristics, leading to a wide spectrum of biological effects. Wozniak et al. obtained five sulfated fucans from brown algae (Scytothamnus australis, Marginariella boryana, Papenfussiella lutea, Splachnidium rugosum and Undaria pinnatifida), four of which could prevent the accumulation of beta-amyloid and AD-like tau in Herpes simplex virus type 1 (HSV1)-infected Vero cells. HSV1 was reported to induces the formation of beta-amyloid, and abnormally phosphorylated tau (P-tau), inducing AD (Wozniak et al., 2015). heparan sulfate (HS) in cell surface mediated Tau spreading in Alzheimer’s disease. fucoidans were expected to compete with HS to bind tau, inhibiting tau spreading. Jin et al. determined the binding abilities of sixty fucoidans/glycans with tau using SPR and AlphaLISA. They found that sulfated galactofucan (SJ-I) and sulfated heteropolysaccharide (SJ-GX-3) inhibited tau-cell interaction and uptake in wild type mouse lung endothelial cell lines, displying strong binding abilities than heparin, suggesting that fucoidans might be potential for inhibiting tau spreading (Jin et al., 2023). Xing et al. displayed that fucoidan from Fucus vesiculosus (FvF) more significantly improved mitochondrial dysfunction, reduced dopaminergic neuron loss, and improved motor deficits than other isolated and purified three different fucoidan species with different chemical structures in an 1-methyl-4-phenyl-1,2,3,6-tetrahydropyridine (MPTP)-induced PD mouse model. Furthermore, the ATP5F1a protein was identified as the key target responsible for alleviating mitochondrial dysfunction (Xing et al., 2023). Yang et al. extracted fucoidans from *Sargassum crassifolium* by warm water, which all showed antioxidant activity dose-dependently in H₂O₂-induced cytotoxicity in rat pheochromocytoma PC-12 cells. Despite the diverse structure, they possessed high and similar neuronal protective properties (Yang et al., 2017). Huang et al. improved the technology of extracting fucoidan from *Sargassum hemiphyllum* via a compressional-puffing process. All extracts showed antioxidant activities and protected SH-SY5Y cells from 6-hydroxydopamine (6-OHDA)-induced apoptosis, among which, phosphorylation of Akt play a vital role against 6-OHDA-induced neurotoxicity (Huang et al., 2017).

Fucoidan could inhibit the release of cytochrome c from the mitochondria to cytosol and caspase dependent apoptosis in beta-amyloid 25-35 (Aβ25-35) and d-galactose induced PC12 cells. Notablely, Fucoidan could regulate the cholinergic system by improving activity of acetylcholine and choline acetyl transferase and attenuating activity of acetylcholine esterase in AD model mice induced by infusion of d-Gal. Furthermore, fucoidan improved antioxidant activity to reduce oxidative stress (Wei et al., 2017). In monocrotophos induced PC12 cells, fucoidan also showed inhibitory activity against cholinergic and monoamine-metabolized enzymes. In addition, fucoidan prevented changes in neurochemicals and latency time of locomotor, learning and memory induced by monocrotophos in *Drosophila melanogaster* (Subaraja et al., 2020). Adella Putri et al., verified that fucoidan possessed good binding affinity with acetylcholinesterase (AChE) with the binding free energy values at −7.4 kcal/mol via a molecular docking approach (Adella Putri et al., 2024). In a transgenic *Caenorhabditis elegans* (*C. elegans*) AD model, fucoidan alleviated Abeta induced paralyzed phenotype through promoting proteolysis. Leading to the reduce the production of reactive oxygen species (ROS) (Wang et al., 2018). Jhamandas et al. used whole-cell patch clamp recording fucoidan on A beta-induced whole-cell currents in acutely dissociated rat basal forebrain neurons and in primary neuronal cultures. The results showed that Fucoidan not only blocks the A beta (25–35) reduction of whole-cell currents in a dose-dependent manner, but also attenuated the downregulation of phosphorylated protein kinase C (Jhamandas et al., 2005). These results demonstrated the potential neuroprotective effects of fucoidan.

Enormous evidence suggest that mitochondria hold a central position in ageing-related neurodegenerative diseases, which regulates cell death, a key feature of neurodegeneration. Mitochondrial DNA mutations and oxidative stress both contribute to ageing. Moreover, an impressive number of disease-specific proteins interact with mitochondria. Thus, therapies targeting energy metabolism or free-radical generation hold great promise (Klemmensen et al., 2024; Zong et al., 2024). Fucoidan was reported to reserve mitochondrial function by regulating PGC-1α/NRF2 pathway in a rotenone-induced rat model (Zhang et al., 2018), and via regulating the AMPK-PGC-1α axis in MPP^+^ induced SH-SY5Y cells (Han et al., 2019). In a 6-hydroxydopamine (6-OHDA) rat model of PD, fucoidan also showed neuroprotective effects. It prevented NADPH oxidases-1 (Nox1)-sensitive oxidative stress and cell damage in both tyrosine hydroxylase (TH)-positive neurons and non-TH-positive neurons. Fucoidan also effectively inhibited nigral microglial activation (Zhang et al., 2014).

In addition, Fucoidan improved the TH protein levels in MPP^+^-induced MN9D cells and in substantia nigra and corpus striatum of MPTP treated mice. Notably, the increase is greater than the level of dopamine and DOPAC, verifying that the dopaminergic terminals are more sensitive than the dopaminergic cell bodies to MPTP toxicity (Luo et al., 2009; Meenakshi et al., 2016). Fucoidan can be purified into several fractions, various fractions of FPS differing in uronic acid and sulfate content showed variable activities. fucoidan fraction sulfated heterosaccharide possessed protective effect against MPP + or - H₂O₂ induced SH-SY5Y cells apoptosis by affecting the PI3K/Akt pathway (Wang et al., 2017; Liu et al., 2020). The high sulfate content of fucoidan may have contributed to its bioactivity. Fucoidan could intervene in many inflammation-related metabolic syndrome, mainly act on several inflammatory process, including modulating inflammation-related gene expression, blocking lymphocyte adhesion and invasion, inhibiting multiple enzymes, and inducing apoptosis (Apostolova et al., 2020; Jayawardena et al., 2022). Fucoidan suppressed tumor necrosis factor-alpha (TNF-alpha)- and interferon-gamma (IFN-gamma)-induced NO production and iNOS expression in C6 glioma cells. Additionally, the activation AP-1, IRF-1, JAK/STAT and p38 mitogen-activated protein kinase (MAPK) was inhibited and the level of scavenger receptor B1 (SR-B1) was improved, suggesting its potential roles for treating inflammatory-related neuronal injury in neurodegenerative disease (Do et al., 2010). Additionally, fucoidan downregulate intracellular ROS and subsequent proinflammatory cytokine release in LPS-activated microglia, NF-κB/MAPK/Akt pathway played an important role (Park et al., 2011; Cui et al., 2012). However, in another study by Murgas et al., fucoidan accelerated the NO production by microglia induced by β amyloid (Aβ) and activated JNK/NF-κB signal pathway (Murgas et al., 2012). The details can be found in the Table 1. Fucoidan is a seaweed polysaccharide with a promising application in the prevention and treatment of NDDs. However, due to its wide source and diverse extraction methods, its molecular weight, sulfate content and structure change, which will affect its pharmacological activity.

**TABLE 1:** Overview of polysaccharides from marine applied in NDDs.

Polysaccharide	Source	Extraction	Characteristics	Disease	Models	Dose	Function	Ref.
λ-carrageenan	*Pseudoalteromonas carrageenovora*	Fermentated by *Pseudoalteromonas carrageenovora* (KCTC 22325)	Including 6.11% of 2.672 kD, 13.7% of 1.319 kD and 456 D, and 1.83% of 185 D	AD	Aβ-induced rats	1% low- and high-MW fucoidan and λ-carrageenan were added into a high-fat diet; Dextrin (1%) as the. negative control; 42 days	Modulated the gut microbiota and their metagenome function	Zhang et al. (2022)
Sulphated Agaran	*Gracilaria cornea*	Protease digestion by papain (60°C, 6 h), followed precipitation with cetylpyridinium chloride	21% yield, 26% sulphate content without antioxidant capacity	PD	6-OHDA- induced rats	15, 30 or 60 μg, int 14 days Rats received 0.9% saline unilateral intrastriatal injection as the control group	Reduced the oxidative/nitroactive stress	Souza et al. (2017)
(Cr-SPs) Polysaccharides	*Chlamydomonas reinhardtii*	Hot water extraction, 80% ethanol precipitation; Anion exchange column Q-Sepharose purification	65% carbohydrates, 34% sulphate, 36% uronic acid, 4% protein	PD	Fibrillation of α-Syn mutants	0.25–1.00 mg/mL	Delayed the conversion of native protein into β-sheet-rich structures	Panigrahi et al. (2019)
Porphyran	*Pyropia haitanensis*	Hot water extraction, 80% ethanol precipitation; Ascorbate and hydrogen peroxide degradation	65.7% total sugar, 13.1% sulfate and 10.4%3,6-anhydro-galactose; with DPPH scavenging activity	AD	Aβ_1-40_ induced mice	75 mg/kg-300 mg/kg, Aβ1-40 solution as the negative control; Donepezil as the positive control; 16 days	Balanced choline acetyltransferase and acetylcholinesterase	(Zhang et al., 2020a)
Dermatan sulfate	*Ascidian Phallusia* nigra	Acetone delipidation, papain for proteolytic digestion, cetylpyridinum chloride precipitation	MW: ∼30 KDa with a polydisperse band	PD	Rotenone induced Neuro-2A cells	0.75–12 μM for 72 h 1.5 μM was the best level	Regulated the activity of the antioxidant enzymes	de Sousa et al. (2020)
Exopolysaccharide	*Keissleriella* sp. YS4108	Cold ethanol precipitation; washed with ethanol, acetone proteins eliminated; DEAE-52 anion-exchange chromatography, Sephacryl S-400 column and Sepharose CL-6B column	Galactose, glucose, rhamnose, mannose and glucuronic acid was 50:8:1:1:0.4	ND	H₂O₂-induced PC 12 cells	100, 200 and 300 μg/mL for 24 h 300 μg/mL was the best level Vitamin E as the positive control	Antioxidant	Sun et al. (2005)
Chitosan	Shrimp source	Tharanathan and Harish Prashanth, 2007, Indian Patent no-231,588, procured from India Sea Foods, Cochin, India	∼20 kDa, 70% Acetylation	PD	*Drosophila melanogaster* exposed to rotenone for 7 days	LMWC concentration 5 mg (LMWC1) and 10 mg (LMWC2) were chosen as the standardized concentration	Enhanced the activity levels of, ETC., enzymes and restored dopamine levels	Pramod Kumar and Harish Prashanth (2020)
	Exoskeletons of crustaceans and the cell walls of fungi and insects	Provided by the Biochemical Engineering College of Beingjing Union University		AD	Aβ_1–42_-induced rat	200, 400, 800,400 mg/kg; huperzine A as a positive control	Reduced cognitive decline	Jia et al. (2016)
		TCI, Tokyo, Japan, C0831		PD	MPTP-induced mice	5 mg/mL/mouse	Reduced acetate levels and inflammation, promoted repair of the intestinal barrier and blood-brain barrier	Wang et al. (2024)
		JAKWANG Co.,Ltd. (Ansung, South Korea)	90% deacetylation	AD	IL-1b and Ab_25–35_ induced human astrocytoma cell line CCF-STTG1	10 μg/mL	Inhibits the production of pro-inflammatory cytokine	Kim et al. (2002)
					H_2_O_2_/FeSO_4_-induced cell death in the NT2 neural cell line	0.1,0.5 w/v	Suppressed Abeta formation	Khodagholi et al. (2010)
Glycosaminoglycan	*Placopecten magellanicus*	Delipidated, proteolysis, captured using strong anion exchange purification using DEAE-anion exchange chromatography	Anticoagulant activity	AD	FRET-based BACE-1 activity assays	4.8 μg/mL	Inhibited BACE-1	Mycroft-West et al. (2023)
	*Portunus pelagicus*	Proteolysis, fractionated by DEAE-Sephacel anion-exchange chromatography utilizing a stepwise sodium chloride gradient	electrophoretic mobility	AD	fluorescence resonance energy transfer inhibition assay		Inhibited BACE-1	Mycroft-West et al. (2019)
ST polysaccharide	*Sargassum thunbergii*	Hot water extraction for 4 h, ethanol precipitation, then separation and purification by anion exchange chromatography	19.70% fucose (Fuc), 14.81% sulfate, 87.98% total sugar, and 18.06% uronic acid, 135 kDa monosaccharides composition was 0.59:0.08:0.31:0.04:0.47:0.08:1.00 (Man:Rha:GlcA:Glc:Gal:Xyl:Fuc)	PD	6-OHDA-induced SH-SY5Y cells	0.05, 0.1, 0.5 mg/mL 0.5 mg/mL was the best level	Well neuroprotective activity	Jin et al. (2018)
Fucoidan	*Fucus vesiculosus*	Defatted in chloroform/methanol for 24 h, digested with papain at 60 °C for 24 h precipitated with cetylpyridinium chloride, then separated by a Q Sepharose Fast Flow anion-exchange column	39.5% ± 1.4% sulfate content; Mw: 103.1 ± 2.8 kDa, The monosaccharide composition is mainly fucose, which accounts for more than 95%	PD	MPTP-induced PD mouse model	Neurons were administered FvF (10 μM) for 48 h. SH-SY5Y cells were administered FvF (5, 10 and 25 μM) for 48 h	Improved mitochondrial dysfunction; Targeted ATP5F1a	Xing et al. (2023)
	*Laminaria japonica*	Hot water extraction	48% total sugar, 28% fucose, and 29% sulfate, fucose: galactose at 1:0.24. The average molecular weight of fucoidan was ∼7 kDa	PD	Rotenone-induced rat model	35, 70, and 140 mg/kg/d for 38 days Rasagiline as the positive control	Regulated PGC-1α/NRF2 pathway	Zhang et al. (2018)
	*Fucus vesiculosus*	Purchased from Sigma Aldrich (St. Louis, MO, USA; Cat# F5631; Batch No. SLBP3196V)	MW: 38.2 kDa; composition: fucose (44.1%), sulfate (26.3%), ash (31.1%), and a little aminoglucose	PD	MPP^+^ induced SH-SY5Y cells	SH-SY5Y cells were exposed to fucoidan (0, 1, 10, 50, and 100 μg/mL) for 24 h or to 50 μg/mL fucoidan for 6, 12, 24, or 48 h 50 μg/mL for 24 h was the best level	Regulated the AMPK-PGC-1α axis	Han et al. (2019)
	*Laminaria japonica*	Hot water extraction	48% total sugar, 28% fucose, and 29% sulfate MW: 7,000 Da Fucose: galactose at 1:0.24	PD	6-OHDA induced rat model	10 or 20 mg/kg, i.p., once daily for 2 or 3 weeks	Prevented oxidative stress and nigral microglial activation	Zhang et al. (2014)
	*Turbinaria decurrens*	Depigmention with acetone followed by hot water extraction	carbohydrate 59.62%, sulfate 26.52%, and Uronic acid 6.3%. MW: 234,000 Da	PD	MPTP treated mice	25 mg/kg body weight for 7 days	Inhibited Aβ accumulation within microglia	Meenakshi et al. (2016)
	*Laminaria japonica*	Hot water extraction, degradation using ascorbate and hydrogen peroxide and purification by DEAE-Sepharose FF exchange chromatography	sulfated heteropolysaccharides; mainly made of fucose, uronic acid and sulfated groups	PD	MPP^+^ or - H₂O₂ induced SH-SY5Y cells	100, 500, 800 μg/mL Modoparas as the positive drugs	Regulated the PI3K/Akt pathway	Wang et al. (2017), Liu et al. (2020)
	*Sargassum crassifolium*	Warm water extraction	2.36% protein, 0.98% lipid, 33.98% ash, and 62.67% carbohydrate Antioxidant activities	PD	H₂O₂-induced PC12 cells	0–2000 μg/mL for 24 h concentration-dependent	Antioxidant	Yang et al. (2017)
	*Sargassum hemiphyllum*	Warm water extraction	53%–68% total sugar 5.66%–7.04% uronic acid contents	PD	6-OHDA induced SH-SY5Y cells	0–500 μg/mL for 24 h concentration-dependent	Anti-apoptosis	Huang et al. (2017)
	*Undaria pinnatifidasporophylls*	trypsin-enzymatic hydrolysis and alcohol grade precipitation	carbohydrates (68.37%), sulfates (21%) and uronic acid (10.89%), protein (0.85%). MW 10.4356 × 10^4^ Da	AD	Aβ 25-35 and d-galactose induced PC12 cells and mice Monocrotophos induced PC12 cells and *Drosophila melanogaster*	65.2–1,000 μg/mL for 48 h or 500 μg/mL fucoidan for 6, 12, 24, 48 or 72 h	Regulate the cholinergic system Antioxidant	Wei et al. (2017), Subaraja et al. (2020)
	brown seaweed	Kangyue Biotech (Xian, China)		AD	Aβ-induced transgenic *Caenorhabditis elegans*	0–2,500 ng/mL	Promote proteolysis	Wang et al. (2018)
				AD	Aβ25-35 induced rat basal forebrain neurons	0.05–10 μM for 48 h	Upregulated PKC	Jhamandas et al. (2005)
	*Undaria pinnatifida*	Treatment by 1% (w/v) H_2_SO_4_ 0.2 M HCl, 2% CaCl_2_ for 6 h in turn		AD	HSV1-infected Vero cells	0–20 μg/mL	Prevented the accumulation of Aβ and AD-like tau	Wozniak et al. (2015)

### 3.2 λ-carrageenan

Carrageenan, a sulphated linear polysaccharide extracted from red seaweeds, was composed of D-galactose residues linked in β-1,4 and α-1,3 galactose-galactose bond (Borsani et al., 2021). Carrageenans have been extensively investigated for various bioactivities such as immunomodulatory activity, antiviral, anticoagulant, antioxidant, and cholesterol-lowering effects (Pangestuti and Kim, 2014). Based on the special negative charge and gelling, it has been used as a viscosity enhancing agent for controlled drug release and prolonged retention and tissue regeneration (Li et al., 2014; Borsani et al., 2021). Low-molecular-weight λ-carrageenan showed improvement on the memory function of rats with an infusion of toxic amyloid-β(Aβ) mainly by increasing the BDNF content and the insulin signaling through enhancing the pSTAT3→pAkt→pGSK-3β pathway. Additionally, regardless of the MW, λ-carrageenan intake could improve gut microbiota to reduce secondary bile acid biosynthesis degradation of toxic compounds, which effects were similar to low-molecular-weight fucoidan, as showed in the increased the abundance of probiotic bacteria *Lactobacillus* and *Akkermentia*. Furthermore, low-molecular-weight fucoidan potentiated hippocampal insulin signaling, increased the expression of ciliary neurotrophic factor and improved glucose tolerance the most (Zhang et al., 2022). Amyloids, with their β-sheet-rich structure, contribute to diabetes and AD. Liposomal nanoformulated iota carrageenan was formulated to effectively disrupt insulin amyloids, which shared structural resemblances with amyloids. The well biocompatibility facilitates its application in prevention and cure of AD (Udayakumar et al., 2024). Limited by the solubility of λ-carrageenan, more studies have been conducted on its oligosaccharides.

### 3.3 Sargassum polysaccharides

Jin et al. extracted several heteropolysaccharides from *Sargassum integerrimum*, *Sargassum maclurei*, *Sargassum naozhouense*, *Spiraea thunbergii*, *S. hemiphyllum* and *Sargassum fusiforme*. The result showed that neuroprotective activities varied according to the structure of the polysaccharides. Although the fragments represented the principal difference between the active and non-active compounds and structures with superoxide-radical scavenging effect were identified, these structures were not associated with neuroprotective effects. Thus, the effect of neuroprotective activities is determined by multiple factors (Jin et al., 2014). *Sargassum thunbergii*, a common intertidal seaweed species, is commonly used as bait and a component of artificial Sargassum beds due to its wide ecological amplitude and high economic and ecological value. Subsequently, Jin et al., prepared two polysaccharides (ST-1 and ST-2) from *S. thunbergii* using anion exchange chromatography. And ST showed well neuroprotective activity. The structure of ST was analyzed by ESI-MS with collision-induced dissociation tandem mass spectrometry (ESI-CID-MS/MS), suggesting that glucuronomannan contained alternating 2-linked Man and 4-linked GlcA, while fucoglucuronan contained 4-linked glucuronan with branched Fuc at C-3. The structure-function relationship need to be further study (Jin et al., 2018).

### 3.4 Polysaccharide from *Chlamydomonas reinhardtii*

α-Synuclein (α-Syn) is an intrinsically disordered presynaptic protein, participates in the progress of PD in an aggregation manner. The sulfated polysaccharides from *Chlamydomonas reinhardtii* (Cr-SPs) were isolated and verified to effectively inhibit α-Syn fibrillation by binding with α-Syn and delaying the conversion of α-helical intermediate into β-sheet rich structures. Cr-SPs are also effective even if onset of α-Syn fibrillation has already started and they also have the ability to dissolve pre-formed fibrils (Choudhary et al., 2018). Fibrillation/aggregation of α-Syn mutants affect specific tertiary interactions essential for stability of the native state. Panigrahi et al., demonstrated Cr-SPs inhibiting effects on fibrillation of α-Syn mutants through efficiently delaying the conversion of native protein into β-sheet-rich structures (Panigrahi et al., 2019). Thus, the finds have substantial therapeutic implications towards PD treatment.

### 3.5 Porphyran

The sulphated polysaccharides drived from marine algae are heterogenic molecules with different biological activities. Polysaccharides from red algae Pyropia haitanensis, named porphyrin, has been reported as a good antioxidant to stimulate immune response in aging mice. Considering its high viscosity, Zhang et al., degraded the polysaccharide and obtained several samples with similar sulfate content to that of natural porphyrin. These composition ameliorated the learning and memory impairment induced by Aβ_1-40_ through increasing choline acetyltransferase (CHAT) activity and decreased acetylcholinesterase (AChE) activity in the cortical and hippocampal tissue, implying that porphyrin might be the potential agents to target aging-related neurodegenerative disease (Zhang Z. et al., 2020). In addition, Wang et al. reported that acetylated porphyran and phosphorylated porphyran, rather than porphyran, significantly antagonized 25 μmol/L 6-OHDA-induced cytotoxicity, but without effect on 75 μmol/L 6-OHDA induced cytotoxicity. However, none could improve mitochondrial transmembrane potential, suggesting their minor neuroprotective effects independent of mitochondria restoration (Wang et al., 2015). An antioxidant degraded porphyran, was regarded as a protective agent against neurotoxicity-induced amyloid β peptide (Aβ) of AD mice. Porphyran significantly ameliorated the learning and memory impairment, balanced ChAT activity and AChE activity in the cortical and hippocampal tissue induced by Aβ1-40 (Zhang Z. et al., 2020).

### 3.6 Other polysaccharides

Souza et al. isolated and obtained a sulphated polysaccharide (SA-Gc) from red marine alga *Gracilaria cornea* presenting a structural 3,6-anhydro-α-l-galactose component. SA-Gc was identified as a polysaccharide of the type agaran, displayed neuroprotective effects through reducing the oxidative/nitroactive stress and modulating the transcription of neuroprotective and inflammatory genes in rat model PD induced by 6-hydroxydopamine (6-OHDA) (Souza et al., 2017). Polysaccharides from *Chlorella pyrenoidosa* (CPS) showed neuroprotective effect in 1-methyl-4-phenyl-1,2,3,6-tetrahydropyridine (MPTP)-induced mouse model of *p*D. The polysaccharides improved motor activity and dopamine expressions, inhibited peripheral immunomodulatory responses in serum and gut. The neuroprotective mechanism might be related to its immunomodulatory action (Chen et al., 2014). Additionally, polysaccharide obtained from *Spirulina platensis* also played a neuroprotective role in MPTP-induced mice, which was likely related to their antioxidative properties (Zhang et al., 2015). Marine microorganisms are considered as efficient producers of biologically active compounds. Sun et al., isolated and authenticated an exopolysaccharide from a marine filamentous fungus-Keissleriella sp. YS4108. The polysaccharide was composed of galactose, glucose, rhamnose, mannose and glucuronic acid, with a mean molecular weight of 1.3 × 10^5^ Da. It showed that the polysaccharide possesses pronounced protective effects against H_2_O_2_-induced cell toxicity through elevating the cell survival and antioxidant activity in a dose-dependent manner (Sun et al., 2005).

## 4 Conclusion and perspective

Polysaccharides, especially marine polysaccharides showed related to regulating the biological processes with anti-apoptosis, anti-inflammatory, antioxidant, anticancer and many other effects. The study of fucoidan and chitosan applied in the treatment of neurodegenerative disorders via inhibiting cell death, inflammation, oxidation and acetylcholinesterase enzymatic activity drew more attention of marine polysaccharides. Now, the reported polysaccharides are mainly from animal and algal sources. A large number of fungi derived polysaccharides need to be further investigated. In addition, Polysaccharides, a heterogeneous class of macromolecules, possess distinct properties depending on their sources and extraction, which determinates their monosaccharide composition and functional groups. The development of polysaccharides as drugs requires a more rigorous standard for extraction and identification, and the polysaccharides containing sulfuric acid groups seem to have better opening value. Furthermore, drug delivery systems, such as chitosan, further expand the application of polysaccharides.
